# Targeting multiple inhibitory receptors to reverse melanoma-induced T cell dysfunction

**DOI:** 10.1186/1479-5876-13-S1-K14

**Published:** 2015-01-15

**Authors:** Hassane M Zarour

**Affiliations:** 1 University of Pittsburgh Cancer Institute Hillman Cancer Center, Pennsylvania, USA

## 

It is now clearly established that dysfunctional/exhausted TA-specific T cells present in peripheral blood and at tumor sites co-express multiple inhibitory receptors. The implications of this important finding are two-fold. First, multiple subsets of TA-specific T cells can be identified in patients with advanced melanoma that exhibit variable levels of T cell dysfunction. Second, this observation supports the implementation of combinatorial therapies aiming at blocking multiple inhibitory pathways to enhance TA-specific immune responses and reverse tumor-induced T cell dysfunction. We have shown that a subset of highly dysfunctional TA-specific CD8+ T cells isolated from patients with advanced melanoma upregulate both PD-1 and Tim-3. PD-1 and Tim-3 blockades strongly enhance TA-specific CD8+ T cell expansion and function. Accordingly, targeting PD-1 and Tim-3 in *vivo* induces melanoma regression in mice. Therefore, the combination of PD-1 and Tim-3 blockade either alone or in combination with cancer vaccines appears to be a promising potent approach to reverse melanoma-induced T cell dysfunction and promote tumor regression in patients with advanced melanoma.

